# Draft genome sequence of a nitrate-reducing, *o*-phthalate degrading bacterium, *Azoarcus* sp. strain PA01^T^

**DOI:** 10.1186/s40793-015-0079-9

**Published:** 2015-10-29

**Authors:** Madan Junghare, Yogita Patil, Bernhard Schink

**Affiliations:** Konstanz Research School of Chemical Biology, University of Konstanz, Constance, D-78457 Germany; Department of Biology, Microbial Ecology, University of Konstanz, Constance, D-78457 Germany

**Keywords:** *Azoarcus sp.* strain PA01^T^, *o*-phthalate degradation, *Rhodocyclaceae*, *Betaproteobacteria*, anaerobic degradation, wastewater treatment plant, pollutant

## Abstract

*Azoarcus sp.* strain PA01^T^ belongs to the genus *Azoarcus*, of the family *Rhodocyclaceae* within the class *Betaproteobacteria.* It is a facultatively anaerobic, mesophilic, non-motile, Gram-stain negative, non-spore-forming, short rod-shaped bacterium that was isolated from a wastewater treatment plant in Constance, Germany. It is of interest because of its ability to degrade *o*-phthalate and a wide variety of aromatic compounds with nitrate as an electron acceptor. Elucidation of the *o*-phthalate degradation pathway may help to improve the treatment of phthalate-containing wastes in the future. Here, we describe the features of this organism, together with the draft genome sequence information and annotation. The draft genome consists of 4 contigs with 3,908,301 bp and an overall G + C content of 66.08 %. Out of 3,712 total genes predicted, 3,625 genes code for proteins and 87 genes for RNAs. The majority of the protein-encoding genes (83.51 %) were assigned a putative function while those remaining were annotated as hypothetical proteins.

## Introduction

Phthalic acid consists of a benzene ring to which two carboxylic groups are attached. There are three isomers of phthalic acid (*o*-phthalic acid, *m*-phthalic acid and *p*-phthalic acid). Phthalic acid esters are widely used as additives in plastic resins such as polyvinyl resin, cellulosic and polyurethane polymers for the manufacture of building materials, home furnishings, transportation apparatus, clothing, and to a limited extent in food packaging materials and medical products [1, 2]. Due to the widespread use of phthalates there has been great concern about their release into the environment [3, 4]. In addition, phthalates and their metabolic intermediates have been found to be potentially harmful to humans due to their hepatotoxic, teratogenic and carcinogenic characteristics [5, 6]. Phthalic acid is also an intermediate in the bacterial degradation of phthalic acid esters [7] as well as in degradation of certain fused-ring polycyclic aromatic compounds found in fossil fuel [8], such as phenanthrene [9], fluorene [10] and fluoranthene [11].

*Azoarcus* sp. strain PA01^T^ (=KCTC 15483) is a mesophilic, Gram-negative, nitrate-reducing bacterium that was isolated from a wastewater treatment plant in Constance, Germany, for its ability to completely degrade *o*-phthalate and a wide range of aromatic compounds. Strain PA01^T^ is also able to grow with a variety of organic substrates including short-chain fatty acids, alcohols, selected sugars and amino acids. These substrates are degraded completely to carbon dioxide coupled to nitrate reduction. The genus *Azoarcus* is comprised of nitrogen-fixing bacteria [12] and known for degradation of aromatic compounds. Currently, this genus consists of nine species with validly published names [13]. These species have been isolated from a wide range of environments, including anoxic wastewater sludge and grass root soil [12]. On the basis of 16S rRNA gene sequence similarity search, the closest relatives of strain PA01^T^ are *Azoarcus buckelii*DSM 14744^T^ (99 % gene similarity) [14, 15] and *Azoarcus anaerobius* (98 %) [16]. *A. buckelii*DSM 14744^T^ was also isolated from a sewage treatment plant for its ability to degrade a wide range of aromatic compounds. But the biochemistry and genetics of anaerobic *o*-phthalate degradation had not been elucidated in detail. Here, we present a summary of the features for *Azoarcus* sp. strain PA01^T^ and its classification, together with the description of the genomic information and annotation.

## Organism information

### Classification and features

*Azoarcus* sp. strain PA01^T^ is a member of the family *Rhodocyclaceae* in the phylum *Proteobacteria*. It was isolated from an activated sewage sludge sample collected (in 2012) from a wastewater treatment plant in Constance, Germany. Enrichment, isolation, purification and growth experiments were performed in anoxic, bicarbonate-buffered, non-reduced freshwater medium containing (g/l); NaCl, 1.0; MgCl_2_ x 6 H_2_O, 0.4; KH_2_PO_4_, 0.2; NH_4_Cl, 0.25; KCl, 0.5; CaCl_2_ x 2 H_2_O, 0.15; NaHCO_3_, 2.5; Na_2_SO_4_, 1 mM. The medium was autoclaved at 121 °C for 25 min and cooled under an oxygen-free mixture of N_2_/CO_2_ (80/20) gas phase. Further, 1 ml trace element solution SL-10 [17], 1 ml selenate-tungstate solution [18] and 1 ml seven-vitamin solution [19] were added. The initial pH of the medium was adjusted to 7.3 ± 0.2 with sterile 1 N NaOH or 1 N HCl. Cultivations and transfer of the strain were performed under N_2_:CO_2_ (80:20) gas atmosphere. The strain was cultivated in the dark at 30 °C. Enrichment cultures were started by inoculating approximately 2 ml of sludge sample in 50 ml freshwater medium (described above) containing 2 mM neutralized *o*-phthalic acid as sole carbon source and 10–12 mM NaNO_3_ as an electron acceptor. Growth was observed after 3–4 weeks of incubation. Enrichment cultures were sub-cultured for several passages with *o*-phthalate as sole carbon source. Pure cultures were obtained in repeated agar (1 %) shake dilutions [20]. Single colonies obtained were retrieved by means of finely-drawn sterile Pasteur pipettes and transferred to fresh liquid medium. The strain was routinely examined for purity by light microscopy (Axiophot, Zeiss, Germany) also after growing the culture with 2 mM phthalate plus 1 % (w/v) yeast extract. For genetic and chemotaxonomic analysis, it was cultivated in the described medium containing 8 mM acetate as a carbon source.

**Fig. 1:**
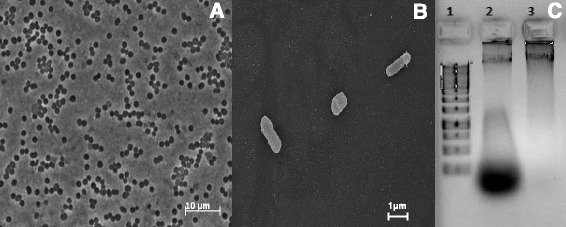
General characteristics of *Azoarcus* sp. strain PA01^T^. **a** Phase contrast micrograph of strain PA01^T^, **b** Scanning electron micrograph of strain PA01^T^, **c** Agarose gel (1 %) electrophoresis of isolated genomic DNA (gDNA) of PA01^T^. Lane 1, 1 kb DNA marker; lane 2, gDNA before RNase treatment; lane 3, high quality gDNA after RNase treatment

*Azoarcus**sp.* strain PA01^T^ is a mesophilic, non-motile, Gram-negative, short rod-shaped bacterium measuring 0.5–0.7 μm (wide), 1.6–1.8 μm (length) (Fig. 1a and b) and divides by binary fission. Growth was observed from 25 °C to 37 °C with an optimum at 30 °C and optimal pH of 7.3 ± 0.2 (Table 1). Strain PA01^T^ grows anaerobically with nitrate on a wide variety of substrates, including *o*-phthalate, benzoate, 3,4-dihydroxy-benzoate, 3-hydroxy-benzoate, 4-hydroxy-benzoate, maltose, fructose, glucose, gluconate, ethanol, 1-butanol, 1-propanol, glycerol, arginine, alanine, malate, pyruvate, succinate, crotonate, propionate, valerate and butyrate. No growth was observed with *iso*-phthalate, *tere*-phthalate, 4-amino-benzoate, resorcinol, methanol, threonine, choline, betaine, formate, citrate, 2-oxoglutarate and oxaloacetate.

**Table 1:** Classification and general features of *Azoarcus sp.* strain PA01^T^ according to the MIGS recommendations [28]

MIGS ID	Property	Term	Evidence code^a^
	Classification	Domain *Bacteria*	TAS [44]
		Phylum *Proteobacteria*	TAS [45]
		Class Betaproteobacteria	TAS [46, 47]
		Order *Rhodocyclales*	TAS [46, 48]
		Family *Rhodocyclaceae*	TAS [46, 49]
		Genus *Azoarcus*	TAS [12]
		Species *Azoarcus* sp.	TAS [12–16]
		Strain: PA01^T^	IDA
	Gram stain	Negative	TAS [12, 15]
	Cell shape	Short rods	IDA
	Motility	Non-motile	IDA
	Sporulation	Not-reported	IDA
	Temperature range	25–37 °C	IDA
	Optimum temperature	30 °C	IDA
	pH range; Optimum	6–8; 7.3 ± 0.2	TAS [15],IDA
	Carbon source	o-phthalate, benzoate, 3 hydroxy-benzoate, 3,4 di-hydroxy-benzoate,, sugars, fatty acids, alcohols, amino acids etc.	IDA
MIGS-6	Habitat	Freshwater, sewage sludge	TAS [12, 15]
MIGS-6.3	Salinity	Not reported	
MIGS-22	Oxygen requirement	anaerobic/aerotolerant	TAS [12, 15]
MIGS-15	Biotic relationship	free-living	NAS
MIGS-14	Pathogenicity	None	IDA
MIGS-4	Geographic location	Constance, Germany	IDA
MIGS-5	Sample collection	2012	IDA
MIGS-4.1	Latitude	47.67° N	IDA
MIGS-4.2	Longitude	9.14° E	IDA
MIGS-4.4	Altitude	397 m	IDA

^a^Evidence codes

*IDA* Inferred from Direct Assay, *TAS* Traceable Author Statement (i.e., a direct report exists in the literature), N*AS* Non-traceable Author Statement (i.e., not directly observed for the living, isolated sample, but based on a generally accepted property for the species, or anecdotal evidence). These evidence codes are from the Gene Ontology project [50]. If the evidence code is IDA, the property was directly observed by one of the authors or an expert mentioned in the acknowledgments

**Fig. 2:**
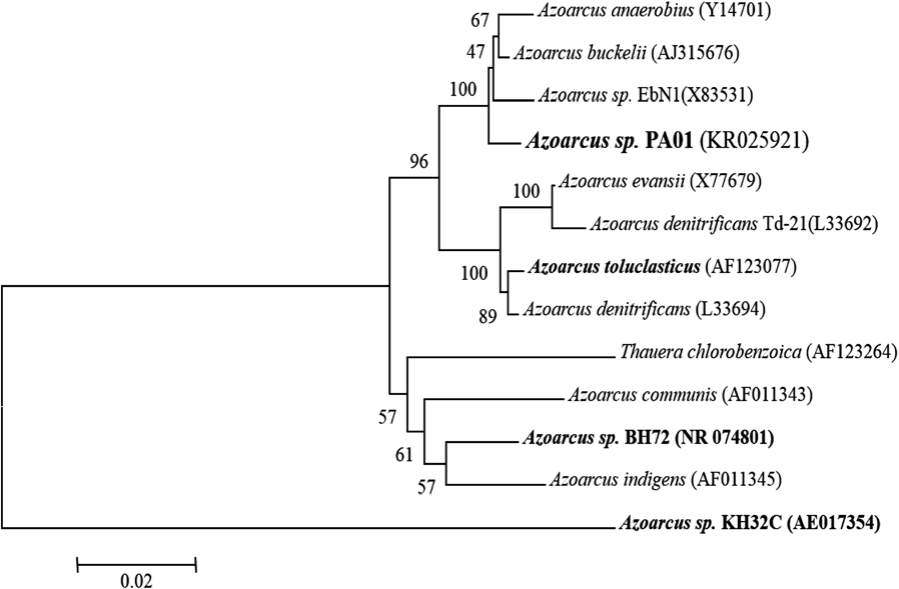
Neighbor-joining phylogenetic tree generated using MEGA4 software package based on 16S rRNA gene sequences. The phylogenetic tree shows the exact position of strain PA01^T^ and the three *Azoarcus spp.* (in bold) whose genome sequence are published, along with other representatives of the genus *Azoarcus*. The corresponding 16S rRNA gene accession numbers are given in parenthesis. Bootstrap values are calculated from 1000 repeats; bar, 0.02 substitutions per nucleotide position

Initial identification and validation of strain PA01^T^ was performed by 16S rRNA gene amplification using a set of universal bacterial primers; 27 F (5′- AGA GTT TGA TCM TGG CTC AG-3′) and 1492R (5′-TAC GGY TAC CTT GTT ACG ACT T-3′) as described [21]. A phylogenetic tree was constructed from the 16S rRNA gene sequence together with the other representatives of the genus *Azoarcus* (Fig. 2) using the MEGA 4 software package [22]. Phylogenetic analysis indicated that strain PA01^T^ belongs to the genus *Azoarcus* and is closely related to *Azoarcus buckelii* (99 %) and *Azoarcus anaerobius* (98 %). Currently, 30 genome sequences are available for the members of the order *Rhodocyclales*. The closest neighbors of strain PA01^T^ whose genome sequence is available are *Azoarcus* sp. strain KH32C [23] and *Azoarcus* sp. strain BH72 [24] and *Azoarcus toluclasticus*ATCC 700605 [25]. The exact phylogenetic position of strain PA01^T^ within the genus *Azoarcus* is shown in Fig. 2 and the 16S rRNA gene sequence of the strain has been deposited to NCBI under accession number KR025921.

#### Chemotaxonomy

Whole-cell fatty acid methyl esters [26] were analyzed by the Identification Service of the Deutsche Sammlung von Mikroorganismen and Zellkulturen (DSMZ, Braunschweig, Germany). The cellular fatty acid pattern of *Azoarcus* sp. strain PA01^T^ is dominated by the presence of an un-saturated branched-chain fatty acid C_16:1_ ω7c/15 iso-2OH (49.6 %) and saturated straight-chain fatty acid C_16:0_ (25.2 %), which have also been reported to be common fatty acids among recently described other species of the genus *Azoarcus* [27, 28]. Other fatty acids include C_18:1_ ω7c (8.8 %), C_17:1_ cyclo (0.82 %), C_16:1_ ω5c (0.68 %), C_14:0_ (0.73 %), C_12:0_ (7.19 %), C_10:0_ 3OH (6.27 %), and C_10:0_ (0.74 %).

## Genome sequencing information

### Genome project history

Strain PA01^T^ was selected for genome sequencing on the basis of its phylogenetic position and its ability to grow on *o*-phthalaet together with numerous aromatic compounds under nitrate-reducing conditions. Genome sequencing was performed at GATC Biotech AG, Konstanz (Germany). High-quality genome draft sequence of *Azoarcus* sp. strain PA01^T^ is listed in the Genomes Online Database of the Joint Genome Institute under project ID Gp0109270 [25]. The *Azoarcus* sp. PA01^T^ whole genome shotgun (WGS) project has been deposited at DDBJ/EMBL/GenBank under the project accession LARU00000000. The version described in this paper has the accession number LARU01000000, and consists of sequences LARU01000001-LARU01000004. The draft genome sequence was released on August 26, 2015. Annotation of the *Azoarcus* sp. strain PA01^T^ genome, was performed by the DOE Joint Genome Institute using microbial genome annotation pipeline state of the art technology [29, 30]. Table 2 presents the project information and its association with MIGS version 2.0 compliance [31].

**Table 2:** Project information

MIGS ID	Property	Term
MIGS 31	Finishing quality	High quality draft
MIGS-28	Libraries used	8–12 kb PacBio library
MIGS 29	Sequencing platforms	PacBio RS
MIGS 31.2	Fold coverage (sequencing depth)	97.42
MIGS 30	Assemblers	HGPA3
MIGS 32	Gene calling method	Prodigal
	Locus Tag	PA01_
	GenBank ID	LARU00000000.1
	GenBank Date of Release	August 26, 2015
	GOLD project ID	Gp0109270
	IMG taxon ID	2596583641
	BIOPROJECT	PRJNA279928
MIGS 13	Source material identifier	KCTC 15483T
	Project relevance	Degradation of aromatic compounds

### Growth conditions and genomic DNA preparation

For the isolation of genomic DNA, cells were grown in one liter medium with 8 mM acetate plus 10–12 mM nitrate. Cells were harvested in the late stationary phase and cell pellet was stored frozen (−20 °C) until DNA preparation. High-molecular-weight genomic DNA was prepared using modified CTAB DNA extraction protocol [32] with some modifications. Chloroform:isoamyl alcohol (24:1) and phenol:chloroform:isoamyl alcohol (25:24:1) steps were repeated twice and RNase treatment was performed for 2 h. Finally, the DNA was dissolved in RNase and DNase-free molecular grade water. Purity, quality and size of the genomic DNA preparation were analyzed by using nanodrop (639 ng/μl, A_260/280_ = 1.84, A_260/230_ = 2.10) and agarose gel electrophoresis (1 % w/v) (see Fig. 1c).

### Genome sequencing and assembly

The genome of *Azoarcus**sp.* strain PA01^T^ was sequenced using a library size of 8–12 kb. Library construction, quantification and sequencing (Pacific Bioscience RS) were performed at GATC Biotech AG (Konstanz, Germany). The final high-quality draft assembly was based on 95,883 reads. The combined libraries provided the 97.42 mean coverage of sequencing depth. Final *de novo* assembly of the genome from the total reads was performed using the PacBio HGAP3 assembly pipeline with default filter parameters. Minimum read length and polymerase read quality was 500 bp and 0.80, respectively. The minimum seed read length was computed automatically and resulted in 5181 bp (length cutoff). The final polished assembly of the sequencing reads yielded 4 linear contigs generating a draft genome size of 3.9 Mb.

### Genome annotation

Annotation was carried out using the DOE-JGI annotation pipeline [30] and genes were identified using Prodigal [33]. The predicted CDSs were translated and used to search the NCBI non-redundant database, UniProt, TIGRFam, Pfam, PRIAM, KEGG, COG and InterPro databases. The tRNAScanSE tool [34] was used to find tRNA genes, whereas ribosomal RNA genes were found by searches against models of the ribosomal RNA genes built from SILVA [35]. Other non-coding RNAs such as the RNA components of the protein secretion complex and the RNase P were identified by searching the genome for the corresponding Rfam profiles using INFERNAL [36]. Additional gene prediction analysis and manual functional annotation was performed within the IMG-ER Platform [37].

## Genome properties

**Fig. 3:**
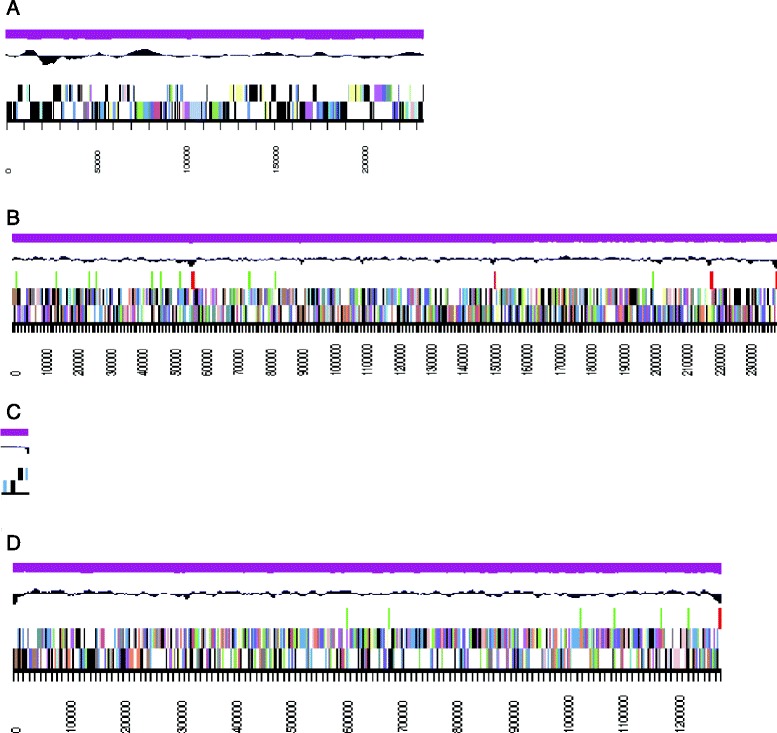
Graphical representation of different scaffolds of the genome of *Azoarcus* sp. strain PA01^T^. **a** Graphical map of *Azoarcus* sp. PA01^T^ genome_PA01_unitig_3_quiver.1. From bottom to top: Genes on forward strand (color by COG categories), Genes on reverse strand (color by COG categories), RNA genes (tRNAs green, rRNAs red, other RNAs black), GC content (black) and GC skew (purple). **b** Graphical map of *Azoarcus* sp. PA01^T^ genome_PA01_unitig_0_quiver.2. From bottom to top: Genes on forward strand (color by COG categories), Genes on reverse strand (color by COG categories), RNA genes (tRNAs green, rRNAs red, other RNAs black), GC content (black) and GC skew (purple). **c** Graphical map of *Azoarcus* sp. PA01^T^ genome_PA01_unitig_2_quiver.3. From bottom to top: Genes on forward strand (color by COG categories), Genes on reverse strand (color by COG categories), RNA genes (tRNAs green, rRNAs red, other RNAs black), GC content (black) and GC skew (purple). **d** Graphical map of *Azoarcus* sp. PA01^T^ genome_PA01_unitig_1_quiver.4. From bottom to top: Genes on forward strand (color by COG categories), Genes on reverse strand (color by COG categories), RNA genes (tRNAs green, rRNAs red, other RNAs black), GC content (black) and GC skew (purple)

The draft genome of *Azoarcus* sp. PA01^T^ is 3,908,301 bp long (with 4 linear contigs, see Fig. 3) with an overall GC content of 66.08 % (Table 3). Of a total 3,712 genes predicted, 3,625 were protein-coding genes, and 87 were RNA genes (15 rRNA genes and 59 tRNA genes); 525 genes without function were identified (pseudogenes). The majority of the protein-coding genes (83.51 %) were assigned a putative function while those remaining were annotated as hypothetical proteins. The properties and the statistics of the genome are summarized in Table 3, the distribution of genes into COGs functional categories is presented in Table 4. One CRISPR region was found in the genome of strain PA01 which is located in proximity to the CRISPR-associated endonucleases (Cas1 and Cas 2) proteins.

**Table 3:** Genome statistics

Attribute	Value	% of Total^a^
Genome size (bp)	3,908,237	100.00 %
DNA coding (bp)	3,511,692	89.95 %
DNA G + C (bp)	2,582,614	66.08 %
DNA scaffolds	4	
Total genes	3,712	100.00 %
Protein coding genes	3,625	97.66 %
RNA genes	87	2.43 %
Pseudo genes	13	0.35 %
Genes with function predictions	3,100	83.51 %
Genes without function prediction	525	14.14 %
Genes assigned to COGs	2,579	69.48 %
Genes with Pfam domains	3,178	85.61
Genes with signal peptides	311	8.38 %
Genes with transmembrane helices	829	22.33 %
CRISPR repeats	1	

^a^The total is based on either the size of the genome in the base pairs or the total number of protein coding genes in the annonated genome

**Table 4:** Number of genes associated with general COG functional categories

Code	Value	% age	Description
J	201	6.93	Translation, ribosomal structure and biogenesis
A	1	0.03	RNA processing and modification
K	141	4.86	Transcription
L	111	3.83	Replication, recombination and repair
B	1	0.03	Chromatin structure and dynamics
D	35	1.21	Cell cycle control, Cell division, chromosome partitioning
V	55	1.90	Defense mechanisms
T	159	5.48	Signal transduction mechanisms
M	195	6.73	Cell wall/membrane biogenesis
N	87	3.00	Cell motility
U	67	2.31	Intracellular trafficking and secretion
O	154	5.24	Posttranslational modification, protein turnover, chaperones
C	250	8.62	Energy production and conversion
G	111	3.83	Carbohydrate transport and metabolism
E	230	7.93	Amino acid transport and metabolism
F	66	2.28	Nucleotide transport and metabolism
H	165	5.69	Coenzyme transport and metabolism
I	187	6.45	Lipid transport and metabolism
P	166	5.73	Inorganic ion transport and metabolism
Q	79	2.73	Secondary metabolites biosynthesis, transport and catabolism
R	210	7.24	General function prediction only
S	146	5.04	Function unknown
-	1113	30.52	Not in COGs

The total is based on the total number of protein coding genes predicted in the genome

## Insight from the genome sequence

*Azoarcus* sp. strain PA01^T^ grows on a wide variety of aromatic compounds (Table 1) linked to nitrate reduction like other bacteria capable of growth via anaerobic degradation of aromatic compounds [38]. In the degradation pathway of most aromatic compounds (including *o*-phthalate), benzoate is a central intermediate and has also been used routinely as the model compound to study the anaerobic degradation of aromatic compounds via the benzoyl-CoA degradation pathway [39]. Annotation of the genome indicated that strain PA01^T^ has key enzymes for the degradation of aromatic compounds such as benzoate. In the past decade, degradation of benzoate through the benzoyl-CoA pathway has been detailed at the molecular level in facultative anaerobes and the phototrophic strictly anaerobic bacteria, i.e. in the denitrifying bacteria *Thauera aromatica* and *Rhodopseudomonas palustris* respectively [40, 41].

Unlike other benzoate and/or aromatic compound degrading bacteria, strain PA01^T^ has the genes for benzoate degradation, which involves a one-step reaction that activates benzoate to benzoyl-CoA by an ATP-dependent benzoate-CoA ligase. The genome of PA01^T^ contains in total two copies of the benzoate-CoA ligase, i.e., benzoate-CoA ligase (EC 6.2.1.25) and benzoate-CoA ligase (EC 6.2.1.25) (locus tag PA01_01819, PA01_03223) which are supposed to be involved in the initial activation of benzoate to benzoyl-CoA. They are located in different positions. These two genes show 68.11 % identity to each other and are also found to be present in the genomes of the other bacteria [23]. The subsequent enzyme of benzoate degradation, benzoyl-CoA reductase is present in one copy with all its four subunits (locus tags PA01_00623, PA01_00625, PA01_00624, PA01_00626) in the genome of strain PA01. The presence of these gene clusters in the genome of *Azoarcus**sp.* strain PA01^T^ provides evidence for the capacity of strain PA01^T^ to degrade aromatic compounds.

Most of the novel biochemistry of the anaerobic metabolism of aromatic compounds has been discovered with nitrate-reducing bacteria in the past two decades [42, 43] and little is known about the biochemistry of phthalate degradation in nitrate-reducing and strictly anaerobic (fermenting and sulfate-reducing) bacteria. We are currently exploring the genome of strain PA01^T^ and the enzymes responsible for *o*-phthalate degradation by using differential proteomics and measuring enzyme activities (unpublished). Thus, the draft genome sequence of strain PA01^T^ provides an opportunity to study the biochemistry of *o*-phthalate degradation into depth.

## Conclusions

*Azoarcus* sp. strain PA01^T^ harbors various genes required for degradation of aromatic compounds (which are normally found in the other aromatic degrading bacteria), e.g., genes for benzoate degradation in the genome of strain PA01^T^. Further, the genome of *Azoarcus* sp. strain PA01^T^ will expands our view to understand the biochemistry of anaerobic degradation of various aromatic compounds, including *o*-phthalate, a priority pollutant. The genome sequence of strain PA01^T^ will provide insight into the putative genes involved in the degradation of all these compounds, mainly *o*-phthalate.

## Abbreviations

PA: Phthalic acid
PAEs: Phthalic acid esters
CoA: Coenzyme A
HGAP3: Hierarchical Genome Assembly Process
CTAB: Cetyl Trimethyl Ammonium Bromide
NCBI: National Center for Biotechnology Information
IMG-ER: Integrated Microbial Genomes-Expert Review
